# Mechanism of Anticancer Activity of Cedrol in Epidermal Carcinoma Cells and Its Validation in 3D Artificial Skin Model

**DOI:** 10.4014/jmb.2601.01050

**Published:** 2026-03-25

**Authors:** You Na Oh, Soojung Jin, EoJin Yu, Somin Jin, Jinsun Jeong, Jung-ha Park, Hee Jung Yun, Hyun Ju Kwon

**Affiliations:** 1 Core-Facility Center for Tissue Regeneration, Dong-eui University, Busan 47340, Republic of Korea; 2 Biohealth Center, RISE Project Group, Dong-eui University, Busan 47340, Republic of Korea; 3 Department of Biopharmaceutics, College of Engineering, Dong-eui University, Busan 47340, Republic of Korea; 4 Anti-Aging Research Center, Dong-eui University, Busan 47227, Republic of Korea

**Keywords:** A431 cells, Anticancer activity, Apoptosis, Cedrol, Epidermal carcinoma 3D skin, G0/G1 phase arrest

## Abstract

In this study, we investigated the anticancer activity of cedrol, a sesquiterpene alcohol, in the human epidermoid carcinoma cell A431 and in epidermal carcinoma 3D skin. Although various physiological activities of cedrol have been reported, the anticancer effect of cedrol in epidermal carcinoma and its molecular mechanisms still remain unclear. Cedrol exhibited significant cytotoxicity in A431 cells in a concentration-dependent manner and reduced the expression of minichromosome maintenance (MCM) proteins. To elucidate the underlying anticancer mechanisms, cell cycle and apoptosis analyses were performed. Cedrol induced dose-dependent G0/G1 phase arrest in A431 cells after 24 h of treatment, accompanied by induction of p53 and p21 and reduction of cyclin E and CDK2. After 48 h of cedrol treatment, apoptosis was observed, as demonstrated by the increase in SubG1 population and Annexin V-positive apoptotic cells using flow cytometry. Cedrol-induced apoptosis was further confirmed by increased expression of the death receptor Fas and the pro-apoptotic protein Bax, decreased expression of the anti-apoptotic protein Bcl-2, release of cytochrome c, and PARP cleavage following caspase activation, indicating apoptosis occurred via both intrinsic and extrinsic pathways. In addition, the cytotoxic effect of cedrol was also observed in an epidermal carcinoma 3D skin constructed using A431 cells, but not in a normal 3D skin constructed using HaCaT cells. Taken together, these findings suggest that cedrol may serve as a novel anticancer therapeutic candidate for epidermal carcinoma and propose that the epidermal carcinoma 3D skin model can be utilized in nonclinical trials.

## Introduction

New cases of skin cancer accounted for approximately 2.9% of all cancer cases in 2022, according to 2024 release data from the Central Cancer Registry [1]. In particular, the incidence of skin cancer has increased sevenfold over the past 20 years, largely due to cumulative ultraviolet (UV) exposure associated with aging [2]. Surgical resection is mainly performed as a cure for skin cancer, and radiation therapy, drug therapy, and chemotherapy are also used. Although resection surgery can be curative at an early stage, its effectiveness is limited in cases of metastasis or recurrence. Conventional chemotherapy also has side effects and can cause drug resistance [3]. Therefore, the development of novel bioactive compounds with selective cytotoxicity to skin cancer tissue and minimal side effects to normal tissue has become very important.

Cedrol is a natural sesquiterpene compound in *Juniperus chinensis*, which is an evergreen conifer of the Cupressaceae family. Cedrol possesses antibacterial [4], sedative [5], autonomic nervous system–regulating [6], anti-inflammatory [7, 8], and skin-whitening effects [9]. According to previous studies, cedrol has been shown to exhibit anticancer activity in various tumors, including colorectal, lung, and liver cancers, primarily by suppression of the cancer-specific markers, minichromosome maintenance (MCM) proteins [4, 10]. However, the anticancer effects of cedrol in epidermal carcinoma cells and its molecular mechanisms have not yet been investigated.

Minichromosome maintenance (MCM) proteins are DNA helicases essential for genomic DNA replication. In eukaryotes, MCM complex consists of MCM2–7, the products of six genes that form a heterohexamer [11, 12]. MCM is a critical protein in cell division, functioning as a key component of multiple checkpoint pathways that regulate S phase entry and progression. [13, 14]. Abnormal overexpression of MCM is consistently observed in various cancer tissues and cancer cell lines, suggesting its potential utility as a biomarker for identifying cancer cells [15-17]. Therefore, identifying modulators that suppress the overexpression of MCM proteins and restore their levels to normal may increase the likelihood of discovering potential anticancer drug candidates [18].

Furthermore, most previous studies evaluating the anticancer activity of cedrol relied on conventional two-dimensional (2D) cancer cell culture systems, which fail to replicate the complex structure and differentiation state of actual human tissue [19, 20]. While these models are cost-effective and easy to use, they often result in inconsistent or poorly predictive of *in vivo* outcomes [21]. Therefore, extensive safety and efficacy evaluations using experimental animals have been conducted to predict the effects of chemicals, including medicines and cosmetics, on the human body. However, the growing ethical concerns about animal use and the limited translational relevance of animal studies to humans have highlighted the need for the development of alternative testing methods [22-24].

Therefore, in this study, we investigated the anticancer activity of cedrol and its underlying molecular mechanisms in human epidermal carcinoma cells using 2D analysis and three-dimensional (3D) artificial skin models. This research provides the first evidence supporting the cedrol-mediated anticancer potential in epidermal carcinoma and emphasizes the usefulness of 3D artificial skin models as a nonclinical evaluation platform.

## Materials and Methods

### Chemicals

Cedrol used in this study was isolated from *J. chinensis* and dissolved in dimethyl sulfoxide (DMSO; Sigma-Aldrich, USA). The cedrol was stored at -20°C and diluted in culture medium prior to use.

### Cell Lines and Culture

Human epidermoid carcinoma A431 and human fibroblast HFF-1 cell lines were purchased from American Type Culture Collection (ATCC, USA). The human keratinocyte cell line HaCaT was obtained from Cell Lines Service (CLS, Germany). A431 and HaCaT cells were cultured in Dulbecco’s modified Eagle’s medium (DMEM; Welgene, Republic of Korea) supplemented with 10% (v/v) fetal bovine serum (FBS; Atlas Biologicals, USA) and 1% penicillin-streptomycin (Cytiva, USA) at 37°C in a humidified environment containing 5% CO_2_.

### Cell Viability Assay and Morphological Study

Cell viability was determined by the water-soluble tetrazolium salt (WST) assay using the EZ-Cytox Cell Viability Assay Kit (DoGenBio, Republic of Korea). HaCaT and A431 cells were seeded in 24-well plates at a density of 2–5 × 10^4^ cells/well and treated with medium containing DMSO (control) or various concentrations of cedrol for 24 and 48 h. EZ-Cytox assay reagent was added to each cell culture well, and the plates were incubated for 30 min at 37°C. The absorbance was measured at 450 nm using a microplate reader (BioTek, USA). The percentage of cell viability was calculated using the following formula: Cell viability (%) = (mean absorbance of tested wells / mean absorbance of control wells) × 100. For morphological analysis, A431 and HaCaT cells were treated with cedrol for 24 and 48 h, and directly imaged using an inverted microscope (Axiovert 40; Carl Zeiss, Germany) equipped with AxioVision software.

### Cell Cycle Analysis

Cell cycle distribution was analyzed using the Muse Cell Cycle Kit (Merck Millipore, Germany) according to the manufacturer's instructions. Cells were seeded in 6-well plates at a density of 1 × 10^5^ cells/well and treated with various concentrations of cedrol for 24 h or 48 h at 37°C. After incubation, approximately 1 × 10^6^ cells were harvested and fixed in cold 70% ethanol for 3 h at –20°C. Fixed cells were resuspended in phosphate-buffered saline (PBS) and mixed with an equal volume of Muse Cell Cycle Reagent. The samples were incubated in the dark for 30 min at room temperature. Cell cycle distribution was then analyzed using Muse Cell Analyzer (Merck Millipore).

### Apoptosis Analysis

Apoptosis was analyzed using the Muse Annexin V and Dead Cell Assay Kit (Merck Millipore) according to the manufacturer's instructions. Cells were seeded in 6-well plates at a density of 1 × 10^5^ cells/well and treated with various concentrations of cedrol at 37°C for 48 h. After incubation, approximately 1 × 10^6^ cells were harvested and resuspended in PBS containing 1% FBS. The cells were then mixed with an equal volume of Muse Annexin V and Dead Cell Reagent and incubated in the dark for 20 min at room temperature. Quantitative analysis of apoptosis was performed using a Muse Cell Analyzer.

### Nuclear Staining with DAPI

A431 cells (5 × 10^4^ cells/well) were plated in 8-well chamber slides and treated with 0.1% DMSO as a vehicle control or with various concentrations of cedrol for 48 h. Cedrol-treated cells were fixed with 4% formaldehyde for 20 min at room temperature and permeabilized with 0.5% Triton X-100 in PBS for 10 min at room temperature. After washing with PBS, the cells were incubated with 1 μg/ml of 4’,6-diamidino-2-phenylindole (DAPI; Sigma-Aldrich, USA) for 10 min, and then washed three times in PBS. Apoptotic nuclei, characterized by chromatin condensation and apoptotic body formation, were examined using a fluorescence microscope (Axio Scope.A1; Carl Zeiss, Germany).

### Western Blot Analysis

Cells were lysed at 4°C for 20 min using whole-cell lysis buffer (Cell Signaling Technology, USA) supplemented with 1 mM phenylmethylsulfonyl fluoride (PMSF) and the supernatant was collected by centrifugation at 14,000 ×*g* for 20 min. In order to extract the cytoplasm protein, the cells were reacted at 4°C for 5 min by adding a cytosolic lysis buffer [20 mM HEPES (pH 7.9), 10 mM KCl, 1 mM EDTA, 10% Glycerol, 0.2% Triton X-100, 1 mM PMSF], and the supernatant was obtained by centrifugation at 1,250 ×*g* for 5 min. After determination of the protein concentration by the Bradford assay, 30-50 μg of protein was separated by 10% SDS-PAGE, transferred to a nitrocellulose membrane (Protran; Sigma-Aldrich, USA), and blocked with 5% skim milk at room temperature for 1 h. The membrane was incubated with specific primary antibodies at 4°C overnight followed by horseradish peroxidase (HRP)-conjugated secondary antibodies (Pierce, USA). Protein bands were visualized using an enhanced chemiluminescence (ECL) detection system (Amersham Life Science, USA) and analyzed with a chemiluminescence image analyzer (Amersham ImageQuant 800; Cytiva, USA). Primary antibodies against p53, p21, FAS, FADD, caspase-3, -8, -9, cleaved caspase-3, -8, -9, Bcl-2, Bax, β-tubulin, lamin B1, cytochrome c, phospho-Rb and PARP were purchased from Cell Signaling Technologies, while the primary antibodies against MCM-2, -3, -6, -7, Rb, CDK2, cyclin E, and actin were purchased from Santa Cruz Biotechnology (USA).

### Artificial Skin Production and Differentiation

For dermal layer fabrication, two fibroblast-containing layers – a lower and an upper layer – were prepared using HFF-1 cells embedded in a collagen type I bioink, according to the manufacturer’s instructions. Briefly, HFF-1 cell pellets were resuspended in 4 mg/ml rat tail collagen I (Corning, USA), and then the pH of the solution was adjusted to 7.6 by adding 15 mM sodium hydroxide. To prepare the lower dermal layer, the bioink containing HFF-1 cells (3.3 × 10^5^ cells/ml) was dispensed into a 12-well transwell insert (pore size: 3 μm; Corning) and gelled for 30 min at 37°C under 5% CO_2_. Subsequently, the upper dermal layer was formed by stacking the bioink containing HFF-1 cells (4 × 10^6^ cells/ml) onto the lower layer. After 24 h of gelation, HaCaT or A431 cells (2 × 10^6^ cells/well) were seeded onto the upper dermal layer and incubated for 2 h without the addition of culture medium. The resulting artificial skin constructs were then immersed in DMEM supplemented with 20% FBS and 0.06 mM calcium ions and cultured for 2 days under liquid–liquid interface (LLI) conditions to stabilize the constructs. Subsequently, DMEM containing 20% FBS, 1.2 mM Ca^2+^, and 50 μg/ml ascorbic acid was added only to the bottom of the wells, and air–liquid interface (ALI) culture was performed for 14 days to induce differentiation and keratinization. The culture medium was replaced every 2 days. Artificial skin experiments were performed using three independent 3D skin constructs. For the viability assay, cedrol was topically applied to the surface of each skin tissue, with doses expressed as mg per tissue.

### Preparation of Tissue Sections

For paraffin embedding, the skin tissue was fixed overnight in 4% formaldehyde and dehydrated by stepwise increasing ethanol concentrations from 70% to 100%. The sample was then cleared in xylene and infiltrated with paraffin for 1 h. Embedding was performed using a paraffin embedding station (Tissue-Tek TEC 5; Sakura, USA), followed by sectioning at 10 μm thickness using a microtome (RM2245; Leica, USA).

### Hematoxylin-Eosin Staining

For hematoxylin–eosin (H&E) staining, paraffin sections were deparaffinized twice with xylene, rehydrated, and rinsed with distilled water. The slides were stained with hematoxylin for 30 sec, washed with water, and immersed in ammonia solution for 1 min. After further washing, the slides were stained with eosin Y for 25 sec. The slides were then dehydrated, immersed in xylene twice for 3 min each, mounted, and observed under an optical microscope (ECLIPSE Ci-L; Nikon, Japan).

### Immunofluorescence Staining for Epidermal Differentiation Markers

Immunofluorescence (IF) staining was performed to detect the expression of epidermal differentiation markers, cytokeratin 10 (CK10) and loricrin, with DAPI used as a nuclear counterstain. Paraffin-embedded tissue sections were deparaffinized, rehydrated, and subjected to antigen retrieval. The slides were blocked at room temperature for 1 h in PBS containing 5% normal goat serum (Sigma-Aldrich) and 5% bovine serum albumin (BSA; Sigma-Aldrich). Sections were then incubated overnight at 4°C with the primary antibodies, followed by incubation at room temperature for 1 h with the appropriate secondary antibodies. After washing, sections were counterstained with DAPI (1 μg/ml) for 5 min, and then mounted using anti-fade mounting medium (Vector Laboratories, USA). Fluorescence signals were observed using a multiphoton confocal microscope (LSM 980 NLO; Carl Zeiss). Primary antibodies against CK10 and loricrin were purchased from Abcam (UK), and Flamma 488- and Flamma 594-conjugated secondary antibodies were obtained from BioActs (Republic of Korea).

### Cytotoxicity Study in Skin Model

Cytotoxicity in the artificial skin model was evaluated using a 3-(4,5-dimethylthiazol-2-yl)-2,5-diphenyl tetrazolium bromide (MTT; Sigma-Aldrich, USA) assay. Olive oil was used as the vehicle control, and cedrol was dissolved in olive oil at various concentrations. After 2 days of treatment at 37°C, 400 μl of MTT solution (0.5 mg/ml) was added to the artificial skin and incubated for an additional 4 h. The supernatant was then removed, and 400 μl of isopropyl alcohol was added to dissolve the formed formazan crystals for 2 h. Absorbance was measured at 540 nm using a microplate reader (Synergy HTX; BioTek, USA).

### Live/Dead Staining of Skin Tissue

The viability of artificial skin tissue was assessed using a Live/Dead Viability/Cytotoxicity Kit (Thermo Fisher Scientific, USA). Differentiated artificial skin tissues were treated with various concentrations of cedrol at 37°C for 2 days, using olive oil as the vehicle control. After washing, samples were stained with 2 μM calcein AM and ethidium homodimer-1 at room temperature for 30 min. Fluorescence signals were observed using a multiphoton confocal microscope (LSM 980 NLO, Carl Zeiss). Skin tissues approximately 100–200 μm thick were imaged by confocal microscopy with 5 μm optical sectioning intervals. The acquired Z-stack images were reconstructed into 3D images using ZEN 3.4 software (Carl Zeiss).

### Statistical Analysis

Data are presented as the mean ± standard deviation (SD) from at least three independent experiments. An unpaired Student’s *t*-test was used to compare each treatment group individually with the vehicle control group using GraphPad Prism version 7 (GraphPad Software Inc., USA). A *p* value < 0.05 was considered statistically significant.

## Results

### Cedrol Inhibits Cell Proliferation and MCM Protein Expression in A431 Cells

**Fig. 1:**
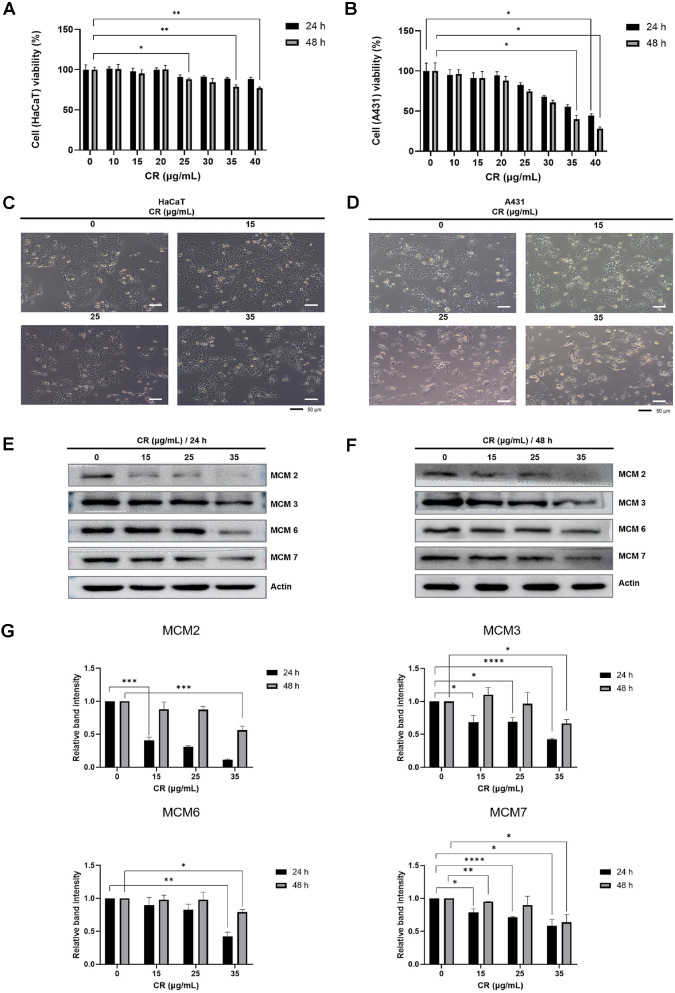
Effect of cedrol on cell growth and MCM protein expression in HaCaT and A431 cells. (**A, B**) HaCaT and A431 cells were treated with cedrol for 24 and 48 h, and cell viability was evaluated using the WST assay. Data are expressed as percentages of vehicle-treated controls ± SD (*n* = 3). *, *p* < 0.05; **, *p* < 0.01 vs. DMSO. (**C, D**) Morphological changes in HaCaT and A431 cells following 48 h of cedrol treatment were observed by light microscopy. Scale bars, 50 μm. (**E, F**) MCM protein expression in A431 cells after cedrol treatment for 24 and 48 h. Actin was used as an internal control. (**G**) The graphs represent the fold change in MCM expression relative to the control group. *, *p* < 0.05; **, *p* < 0.01; ***, *p* < 0.005, ****, *p* < 0.001 vs. DMSO.

To investigate the effect of cedrol on cell viability in normal human keratinocytes and epidermal cancer cells, the human keratinocyte cell line HaCaT and the human epidermoid carcinoma cell line A431 were treated with cedrol, and the cytotoxicity was evaluated using the WST assay. The results showed that cedrol did not induce cytotoxicity in HaCaT cells (Fig. 1A). In contrast, the viability of epidermal cancer cell A431 decreased in a cedrol concentration-dependent manner (Fig. 1B). In particular, after 24 h of cedrol treatment, cell proliferation was inhibited in A431 cells starting at 25 μg/ml. At 48 h of treatment, the cell survival rate decreased by 72% at the highest cedrol concentration (40 μg/ml) (Fig. 1B).

To examine whether cedrol induces morphological alterations in normal keratinocytes and epidermal carcinoma cells, each cell type was treated with various concentrations of cedrol, and changes in cell morphology were observed using phase-contrast microscopy. As shown in Fig. 1C, HaCaT cells maintained clustered growth even at 35 μg/mL of cedrol. In contrast, A431 epidermal carcinoma cells exhibited a gradual decrease in cell density with increasing cedrol concentrations, indicating inhibited cell proliferation (Fig. 1D). Notably, starting at 25 μg/ml of cedrol, A431 cells showed a distinct morphological change, transitioning from a clustered growth pattern to an irregularly dispersed single-cell morphology (Fig. 1D).

Next, to determine whether cedrol treatment alters MCM protein expression in A431 cells, Western blot analysis was performed. As shown in Fig. 1E, 1F, and 1G, cedrol treatment resulted in a concentration-dependent decrease in the expression of MCM proteins (MCM2, MCM3, MCM6, and MCM7) compared with the control group at both 24 and 48 h.

### Cedrol Arrests the Cell Cycle of A431 Cells at G0/G1 Phase

**Fig. 2:**
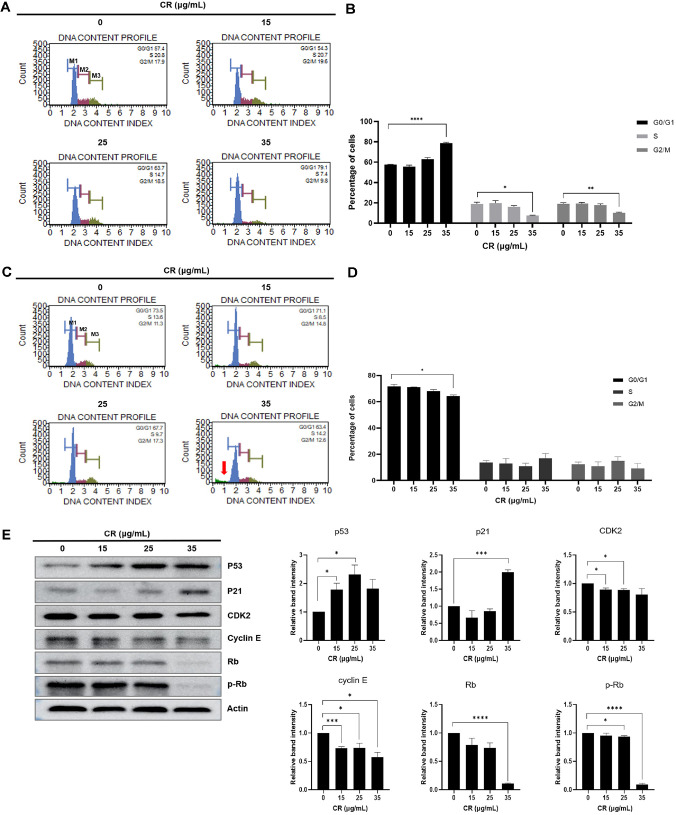
G0/G1 arrest and SubG1 accumulation in cedrol-treated A431 cells. A431 cells were cultured and treated with cedrol for 24 h (**A, B**) and 48 h (**C, D**). (**A, C**) Cell cycle profiles were evaluated using a Muse Cell Cycle Kit with a Muse Cell Analyzer. The arrow indicates the subG1 population of cedrol-treated A431 cells. M1, G0/G1 phase; M2, S phase; M3, G2/M phase. (**B, D**) Quantitative data for cell distribution are shown. Results are expressed as percentages of the vehicle-treated control ± SD of three independent experiments. *, *p* < 0.05, **, *p* < 0.01, ****, *p* < 0.001 vs. DMSO-treated cells. (**E**) Modulation of G0/G1 checkpoint-related protein expression in A431 cells after 24 h of cedrol treatment. Actin was used as an internal control. The graphs represent the fold change in G0/G1 checkpoint-related protein expression relative to the control group. *, *p* < 0.05; **, *p* < 0.01; ***, *p* < 0.005, ****, *p* < 0.001 vs. DMSO.

After treatment of A431 cells with various concentrations of cedrol for 24 and 48 h, cell cycle distribution was analyzed by flow cytometry using propidium iodide (PI) staining. As shown in Fig. 2A and 2B, 24 h of cedrol treatment increased the proportion of cells in the G0/G1 phase from 57.4% to 79.1% in a concentration-dependent manner. In contrast, the S phase population decreased from 20.8% in the control group to 7.4% at the highest cedrol concentration (35 μg/ml), and the G2/M phase population decreased from 17.9% to 9.8%. These results indicate that cedrol induces G0/G1 cell cycle arrest in A431 cells. Furthermore, analysis of cell cycle distribution after 48 h of cedrol treatment revealed a marked increase in the SubG1 population (Fig. 2C and 2D).

To further elucidate the molecular mechanism of cedrol-induced G0/G1 arrest in A431 cells, we examined the expression levels of cell cycle regulatory proteins associated with the G0/G1 checkpoint by Western blot analysis. As shown in Fig. 2E, 24 h of cedrol treatment caused a concentration-dependent increase in the expression of the tumor suppressor protein p53 and the cyclin-dependent kinase inhibitor p21. In contrast, the expression levels of cyclin-dependent kinase 2 (CDK2) and cyclin E were significantly reduced following cedrol treatment. Consequently, the expression levels of retinoblastoma (Rb) protein and phosphorylated Rb (p-Rb) were also decreased (Fig. 2E). These findings further support that cedrol induces G0/G1 cell cycle arrest in A431 cells.

### Cedrol Induces Apoptosis in A431 Cells

**Fig. 3:**
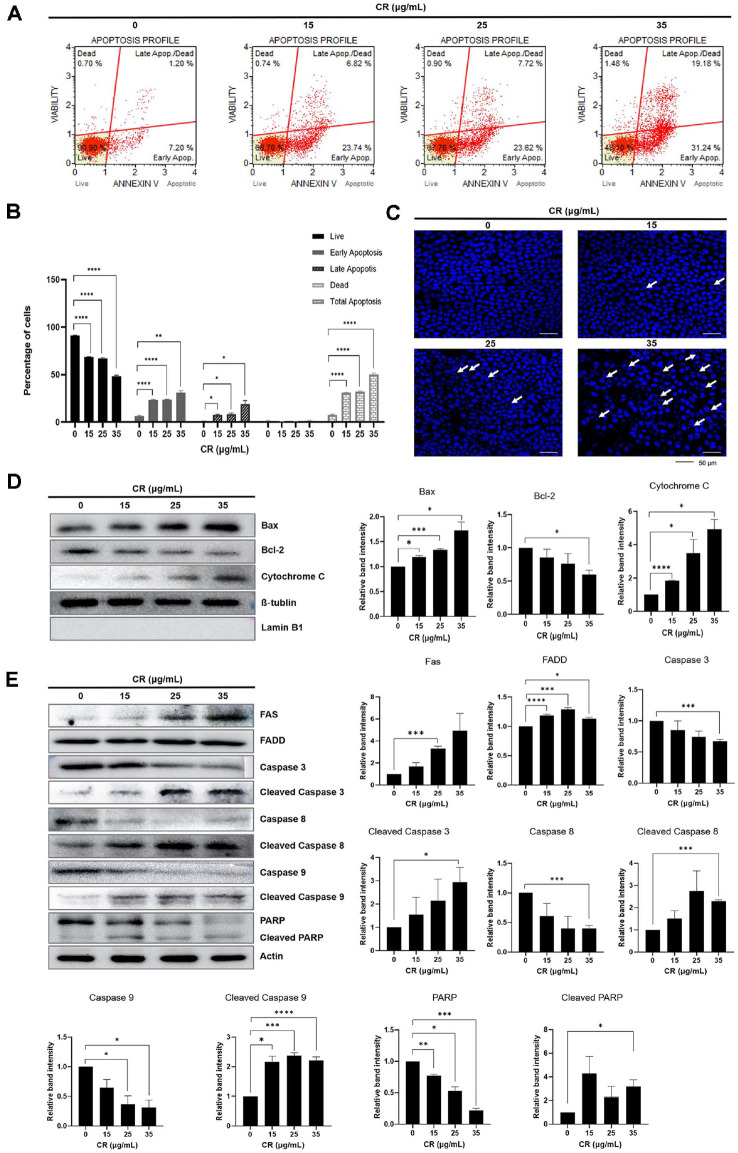
Apoptosis induction in cedrol-treated A431 cells. (**A**) A431 cells were cultured and treated with cedrol for 48 h. Apoptosis profiles were analyzed using a Muse Annexin V and Dead Cell Assay Kit with a Muse Cell Analyzer. (**B**) Quantitative data for cell distribution are shown. UL, dead cells; UR, late apoptotic/dead cells; LL, live cells; LR, early apoptotic cells. Results are expressed as percentages of the vehicle-treated control ± SD of three independent experiments. *, *p* < 0.05, **, *p* < 0.01, ****, *p* < 0.001 vs. DMSO-treated cells. (**C**) DAPI staining showing apoptotic morphological changes of A431 cells after cedrol treatment. Arrows indicate the chromatin condensation. Scale bars, 50 μm. (**D**) Modulation of apoptosis-related protein expression (cytosolic fraction) in A431 cells after 48 h of cedrol treatment. β-tubulin and lamin B1 were used as internal controls for cytoplasmic and nuclear protein, respectively. The graphs represent the fold change in apoptosis-related protein expression relative to the control group. *, *p* < 0.05; **, *p* < 0.01; ***, *p* < 0.005, ****, *p* < 0.001 vs. DMSO. (**E**) Modulation of apoptosis-related protein expression (whole cell lysate) in A431 cells after 48 h of cedrol treatment. Actin was used as an internal control. The graphs represent the fold change in apoptosis-related protein expression relative to the control group. *, *p* < 0.05; **, *p* < 0.01; ***, *p* < 0.005, ****, *p* < 0.001 vs. DMSO.

To determine whether this increase in the SubG1 population after 48 h of cedrol treatment was associated with apoptosis, Annexin V assays were performed on A431 cells treated with various concentrations of cedrol for 48 h. As shown in Fig. 3A and 3B, the total apoptotic cell population (Annexin V^+^) increased in a dose-dependent manner from 8.4% to 50.4% at the highest concentration. At 35 μg/ml cedrol, early apoptotic cells (Annexin V^+^/7-AAD^-^, 31.24%) were more abundant than late apoptotic cells (Annexin V^+^/7-AAD^+^, 19.18%).

To investigate nuclear morphological changes associated with cedrol-induced apoptosis, A431 cells were treated with indicated concentrations of cedrol, followed by DAPI staining. As shown in Fig. 3C, the number of condensed chromatin bodies (apoptotic bodies) increased in a dose-dependent manner compared with the control group.

Western blot analysis was then performed to examine changes in apoptosis-related proteins following cedrol treatment. The expression of the death receptor Fas was upregulated, and the levels of the pro-apoptotic Bcl-2 family protein Bax and cytosolic cytochrome c increased. Conversely, levels of the anti-apoptotic protein Bcl-2 decreased (Fig. 3D). These changes resulted in activation of the caspase cascade, as demonstrated by increased levels of cleaved caspase-3, caspase-8, and caspase-9. Ultimately, cleavage of PARP was observed, confirming that cedrol induces apoptosis in A431 cells (Fig. 3E).

### Histological Characteristics and Differentiation Marker Expression of Artificial Skin Models

**Fig. 4:**
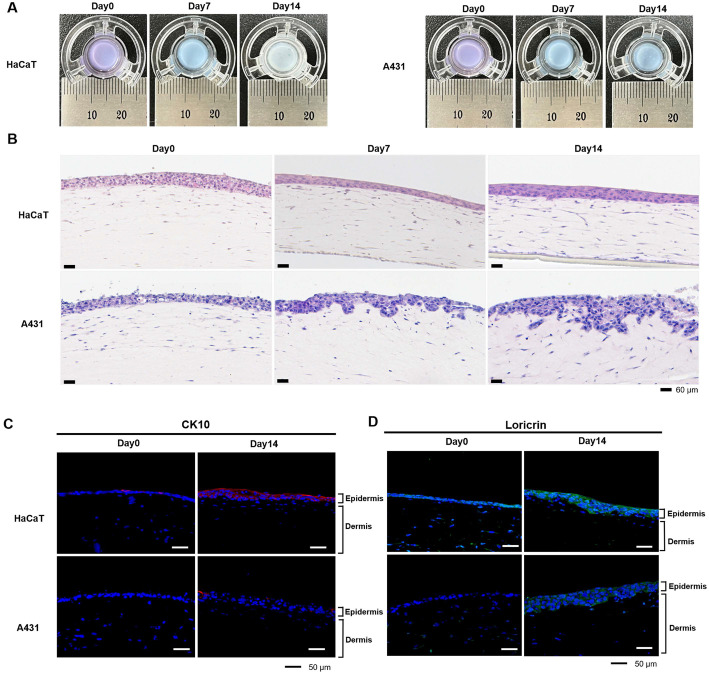
Fabrication of artificial skin and histological analysis. (**A**) 3D Artificial skin models were constructed using HaCaT or A431 cells together with HFF-1 cells and cultured under differentiation conditions for 14 days. (**B**) Histological analysis by H&E staining. Artificial skin models generated with HaCaT or A431 cells were examined for histological properties at each differentiation period (days 0, 7, 14). Scale bars, 60 μm. (**C, D**) Immunofluorescence staining of paraffin sections of artificial skin using keratinocyte differentiation markers, CK10 (red) and loricrin (green). DAPI was used as a nuclear counterstain. Scale bars, 50 μm.

To evaluate the anticancer activity of cedrol against epidermal carcinoma cells in a three-dimensional (3D) artificial skin system, we established normal 3D skin model and epidermal carcinoma 3D skin models. To induce differentiation and keratinization of the epidermal layer in each model, the 3D constructs were first stabilized by culturing for 2 days under LLI conditions, followed by ALI culture for 14 days (Fig. 4A). The histological characteristics of the skin models at days 0, 7, and 14 after ALI culture were examined by H&E staining of tissue sections. As shown in Fig. 4B, the normal 3D skin composed of epidermal keratinocyte HaCaT cells exhibited a slight decrease in epidermal thickness as differentiation progressed, with a more compact and stratified epidermal layer and a clear boundary between the epidermis and dermis. In contrast, the artificial skin constructed with epidermal carcinoma A431 cells failed to form a dense and stratified epidermal structure. Moreover, A431 cells invaded the dermal layer, resulting in a disorganization of epidermal structure and hyperproliferative features (Fig. 4B).

Furthermore, the expression patterns of the differentiation markers cytokeratin 10 (CK10) and loricrin were analyzed by immunofluorescence staining of artificial skin tissues at day 0 and day 14 after ALI culture. As shown in Fig. 4C, CK10 expression was markedly increased in the 14-day post-ALI cultured tissue of normal 3D skin, whereas no significant CK10 expression was observed in the epidermal carcinoma 3D skin. Similarly, loricrin expression was significantly increased in the terminally differentiated regions of the epidermal layer in the normal 3D skin, but was scarcely detected in the epidermal carcinoma 3D skin (Fig. 4D).

Overall, these results confirm that epidermal differentiation was successfully achieved in the normal 3D skin, whereas the epidermal carcinoma 3D skin maintained an undifferentiated state and hyperproliferative phenotype.

### Cedrol-Induced Cytotoxicity in the Epidermal Carcinoma 3D Skin

**Fig. 5:**
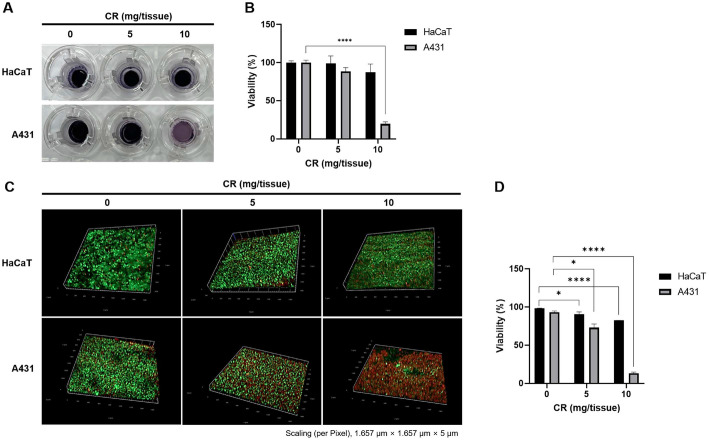
Cytotoxicity of cedrol in artificial skin. (**A**) Artificial skin was treated with various concentrations of cedrol for 48 h. Cytotoxic effect of cedrol was determined by the MTT assay. (**B**) Results are expressed as percentages of the vehicle-treated control ± SD of three independent experiments. ****, *p* < 0.001 vs. olive oil-treated cells. (**C**) Cell viability assessed by fluorescent staining using Live/Dead Viability/Cytotoxicity Kit; live cells (green) and dead cells (red). Scaling (per Pixel): 1.657 μm × 1.657 μm × 5 μm. (**D**) Quantitative analysis of the live/dead assay was performed using ZEN 3.4 software. Cell viability (%) was calculated by determining the proportion of live cells relative to the total cell population. *, *p* < 0.05; ****, *p* < 0.001 vs. DMSO.

To evaluate the cytotoxic effects of cedrol in differentiated artificial skin, an MTT assay was performed after treating the skin with indicated concentration of cedrol. After 3 h of incubation with MTT reagent, the amount of formazan generated in the epidermal carcinoma 3D skin was found to be significantly lower than that in the normal 3D skin (Fig. 5A). After dissolving the formazan crystals in isopropyl alcohol and measuring the absorbance at 540 nm, the survival rate of normal 3D skin tissue at 10 mg/tissue cedrol was 87.6%, whereas that of the epidermal carcinoma 3D skin tissue was 19.6% (Fig. 5B).

We further performed live/dead staining on the differentiated artificial skin models to qualitatively assess cell viability following cedrol treatment. In the normal 3D skin containing HaCaT cells, no notable changes in the distribution of live cells (green) were observed after cedrol treatment (Fig. 5C). In contrast, in the epidermal carcinoma 3D skin containing A431 cells, the distribution of dead cells (red) was markedly increased in a concentration-dependent manner (Fig. 5C). The viability of normal 3D skin tissue at 10 mg/tissue cedrol was 82.6%, whereas that of the epidermal carcinoma 3D skin tissue was 13.2% (Fig. 5D). These results demonstrate that cedrol exhibits minimal cytotoxic effects on normal 3D skin while selectively reducing cell viability in epidermal carcinoma 3D skin, suggesting its potential as an anticancer agent against epidermal carcinoma 3D skin.

## Discussion

In this study, cedrol-mediated anticancer activity and its underlying molecular mechanisms in human epidermal cancer cells were investigated using both conventional 2D cell-based assays and a human cell-based 3D artificial skin model. Although the anticancer activity of cedrol has been reported in several cancer types, its effect on epidermal carcinoma cells has not been previously investigated. To the best of our knowledge, this study is the first to elucidate the anticancer effects of cedrol in epidermal carcinoma by using both 2D and 3D experimental systems.

MCM proteins act as helicases that form the pre-replicative complex to facilitate DNA replication in eukaryotes and have been reported to be markedly overexpressed in various types of cancer compared to normal tissues [25-27]. Consequently, MCM proteins have attracted attention as potential target molecules for the development of novel anticancer therapeutics [28, 29]. Cedrol is known to suppress the expression of MCM proteins in colorectal and liver cancer cells. In the present study, we found that cedrol treatment similarly inhibited MCM protein expression in A431 cells, suggesting that MCM proteins may serve as a common molecular target of cedrol across multiple cancer types.

In the normal cell cycle, progression from G1 to S phase requires the formation and activation of the cyclin E/CDK2 and cyclin D/CDK4 complexes, leading to the sustained phosphorylation of Rb protein and then releasing the E2 promoter-binding factor (E2F) transcriotion factor from Rb. Subsequently, the expression of various genes necessary for S phase entry is upregulated, allowing the cell cycle to progress into the S phase [30]. However, when cells experience damage such as DNA damage, cellular aging, or stress signaling, the cell cycle is arrested. If the damage persists or becomes severe without proper repair, apoptosis is induced to eliminate the damaged cells [31]. DNA damage and cellular stress increase p53 expression, which ultimately upregulates p21. p21 inhibits cell cycle progression by binding to cyclin-CDK complexes and proliferating cell nuclear antigen (PCNA), thereby blocking DNA replication [32]. In the present study, cedrol treatment induced G0/G1 phase arrest along with increased expression of p53 and p21 and downregulation of CDK2, cyclin E, and phosphorylated Rb. Although this study did not directly prove the causal relationship between MCM suppression and p53/p21 pathway activation, previous reports have suggested that MCM dysregulation are associated with replication stress and checkpoint activation [18,25]. Therefore, based on our experimental observations and existing literature, we propose that reduction in MCM protein expression induced by cedrol may contribute to p53/p21-dependent cell cycle arrest in epidermal carcinoma cells.

Apoptosis, one of the major mechanisms for eliminating cancer cells, is induced through intrinsic and extrinsic pathways [33, 34]. The intrinsic pathway is triggered by internal stress signals, such as DNA damage and oxidative stress, which induce an imbalance between pro- and anti-apoptotic Bcl-2 family proteins. This imbalance increases mitochondrial outer membrane permeability and causes loss of mitochondrial membrane potential, leading to the release of cytochrome c and subsequent activation of caspase-9 [35, 36]. The extrinsic pathway is initiated by the binding of death ligands, such as FasL and tumor necrosis factor (TNF), to their corresponding cell surface death receptors Fas and TNFR, respectively, leading to the activation of caspase-8 [37, 38]. Both the intrinsic and extrinsic pathways activate the caspase cascade, in which activated caspase-8 and caspase-9 subsequently activate caspase-3. Activated caspase-3 then plays a crucial role in the execution of apoptosis by cleaving substrate proteins such as poly (ADP-ribose) polymerase (PARP) [39-41].

In addition to cell cycle arrest, our findings demonstrated that cedrol induces apoptosis in epidermal carcinoma cells by activation of both intrinsic and extrinsic pathways. Cedrol treatment exhibited the reduction of Bcl-2 expression, increase of Bax expression, enhancement of cytochrome c release, and activation of caspase-9, suggesting involvement of the intrinsic pathway. Simultaneously, caspase-8 activation was observed following upregulation of Fas and FADD, suggesting potential activation of the extrinsic pathway.

Collectively, these results suggest that cedrol may exert anticancer effects by inhibiting MCM protein expression, thereby activating the p53/p21 pathway, inducing G0/G1 arrest, and ultimately triggering apoptotic cell death.

Next, we verified the anticancer efficacy of cedrol through nonclinical experiments using layered structure artificial skin models, including normal 3D skin and epidermal carcinoma 3D skin. The epidermis is composed of stratified layers formed through the differentiation of basal keratinocytes, which proliferate in the basal layer and migrate upward to form the spinous layer, granular layer, and ultimately the stratum corneum [42]. The degree of epidermal differentiation can be evaluated by the expression of specific molecular markers, including CK10 and loricrin. CK10, one of the structural proteins, is broadly expressed in suprabasal layers, including the spinous and granular layers of the epidermis [43, 44]. Loricrin is a marker for the late stage of epidermal differentiation predominantly expressed from the middle of the spinous to the granular layer, and is associated with terminal keratinocyte differentiation [45].

Cutaneous squamous cell carcinoma (cSCC) is classified into well-differentiated cSCC and poorly-differentiated cSCC based on the degree of cellular differentiation [46]. Well-differentiated cSCC generally exhibits slow growth and a low risk of metastasis, while poorly differentiated cSCC is considered a high risk subtype with increased rates of recurrence and metastasis [47]. In poorly-differentiated cSCC, the expression of the key epidermal differentiation markers has been reported to be significantly reduced or nearly absent [48]. Additionally, a high mitotic count was observed, and these features have been reported to be associated with higher malignancy, invasiveness, and metastatic potential [49].

In this study, normal 3D skin and epidermal carcinoma 3D skin were generated using HaCaT and A431 cells, respectively, and the degree of epidermal differentiation and keratinization was confirmed by H&E and IF staining. IF staining of tissue at day 14 after ALI differentiation culture revealed that CK10 and loricrin were expressed in the epidermal layer of normal skin tissue, consistent with the differentiation stage. In contrast, there was no significant expression of CK10 or loricrin in the epidermal carcinoma 3D skin. Therefore, these histological and molecular characteristics suggest that the epidermal carcinoma 3D skin model used in this study reproduces the key features of poorly-differentiated cSCC, supporting its relevance as a nonclinical experimental model.

To verify the anticancer efficacy of cedrol through nonclinical testing, toxicity assessments were conducted using both normal 3D skin and epidermal carcinoma 3D skin. The results showed that cedrol induced remarkable concentration-dependent cell death in the epidermal carcinoma 3D skin, whereas it had no significant effect on normal 3D skin. These findings are consistent with those obtained from 2D cell-based experiments.

Collectively, our findings indicate that cedrol acts as a novel anticancer agent against epidermal carcinoma by suppressing MCM protein expression, inducing G0/G1 cell cycle arrest, and triggering apoptosis. Furthermore, this study provides a foundation for the development of effective nonclinical testing platforms to evaluate the efficacy and safety of anticancer agents using a 3D epidermal carcinoma skin model as alternatives to animal testing.
